# Parents’ knowledge, attitudes and beliefs regarding sun protection in children: a qualitative study

**DOI:** 10.1186/s12889-018-5091-8

**Published:** 2018-02-01

**Authors:** Zoe Littlewood, Sheila Greenfield

**Affiliations:** 1 College of Medical and Dental Sciences, University of Birmingham, Edgbaston, Birmingham, B15 2TT UK; 2 Institute of Applied Health Research, College of Medical and Dental Sciences, Murray Learning Centre, University of Birmingham, Edgbaston, Birmingham, B15 2TT UK

**Keywords:** Children, Parents, Sun-related behaviours, Sun protection, Qualitative research, Attitudes, Belief, Knowledge

## Abstract

**Background:**

Childhood is a critical period for sun protection, when the skin is particularly susceptible to the carcinogenic effects of ultraviolet radiation. Children are dependent upon parents to implement sun protective measures. Existing qualitative research exploring parents’ attitudes and beliefs underpinning children’s sun protection is from Australia, which has the highest melanoma incidence rates globally, and thus benefits from widespread sun protection awareness campaigns. Parents’ sun protective behaviour may, therefore, differ between Australia and the UK.

This study investigates the topic in a UK context, using qualitative methodology to gain detailed insights into a relatively under-researched area. The aim of the study was to explore parents’ knowledge and understanding of sun protection in children, and factors that motivate and challenge them in this area. Finally, it aimed to determine if and how ethnicity and skin type influence these attitudes and beliefs.

**Methods:**

Twenty-two semi-structured individual interviews were carried out with parents of children aged 5 years or younger, recruited from local nurseries. Transcripts were analysed using thematic analysis.

**Results:**

Four overarching themes emerged, each incorporating two to three sub-themes. ‘Attitudes towards children’s sun protection’ refers to the fact that parents considered sun protection to be important for children, a finding which was consistent between different skin types. ‘Sun protection practices’ brings together several protective behaviours adopted in children and, to a lesser degree, in parents, and their associated disadvantages. ‘Sun safety knowledge’ refers to parents’ awareness of the risks of sun exposure and the need for protection, and illustrates where gaps in knowledge exist, such as regarding the need for vitamin D, and the importance of vigilant sun protection even in the UK. Finally, ‘motivating and facilitating factors’ highlights motivations for sun protection in children, and factors that facilitate it in practice.

**Conclusion:**

This study found parents to be motivated and concerned about children’s sun protection, irrespective of children’s ethnicity, and aware of appropriate protective behaviours. It indicates key challenges which could be targeted in future campaigns in order to improve sun protection in children and reduce uncertainty and anxiety regarding sun safety amongst parents.

**Electronic supplementary material:**

The online version of this article (10.1186/s12889-018-5091-8) contains supplementary material, which is available to authorized users.

## Background

It is well-established that overexposure to ultraviolet (UV) radiation is a major risk factor for melanoma skin cancer [1]. Protecting the skin from the harmful effects of the sun, the principal source of UV radiation, is therefore a key public health priority in the prevention of this disease, the incidence of which has increased by 119% in the UK since the early 1990s and continues to rise [2].

Despite the implementation of several campaigns throughout the last decade [3], evidence suggests that sun protection behaviour in the UK is still inadequate. In a recent survey, it was reported that 50% of individuals had experienced sunburn at least once in the previous year [4].

Childhood is a particularly crucial period for sun protection. Ecological studies demonstrate that sun exposure during early life, especially childhood and adolescence, is a strong determinant of future risk of melanoma, suggesting that the skin at this age is at increased susceptibility to the carcinogenic effects of ultraviolet radiation [5–7]. It is thus recommended that children’s skin be protected from the sun from March until October in the UK through the use of sunscreen, shade, suitable hats and clothing [8]. Given that young children are unable to understand the consequences of excess sun exposure or adopt sun protective practices independently, they are dependent upon their parents or carers to implement such protection.

Despite the importance of sun protection in children, a Met Office survey has highlighted a shortfall in parents’ knowledge and protective practices [9]. Of 1000 parents with children aged 11 and under, 7% failed to understand the strong link between UV radiation and cancer, 21% revealed that their children would typically be visibly burnt before protective measures were taken and 40% of children had experienced sunburn in the past 2 years. Evidently, there remains considerable room for improvement in children’s sun protection.

Adolescents’ sun protection behaviours are difficult to target due to negative attitudes towards the use of sun protection, significant peer influences and a common desire to be tanned [10]. With this in mind, it has been suggested that targeting children may be more achievable and confer longer lasting benefits, as health behaviours and habits established during childhood are often seen to continue into adulthood [6].

The majority of literature regarding children’s sun protection is based on population based surveys and questionnaires. These show considerable awareness amongst parents about sun exposure, skin cancer, and appropriate protection [11, 12]. Quantitative literature also demonstrates how parents’ knowledge and attitudes influence these protective behaviours. Parents with greater awareness of the risks of sun exposure more effectively protect their children [13–16], whilst for those with positive attitudes towards tanning, the converse is true [10, 11, 13, 17].

Qualitative methods allow a richer understanding to be brought to this matter, accommodating the complexities and nuances of parents’ beliefs and attitudes in a way that quantitative methods are unable to do [18]. Qualitative research with parents on this topic from the UK is limited to a summary of findings from focus groups with mothers carried out on behalf of Cancer Research UK [19]. The study itself is unpublished, however the summary illustrates that mothers are generally receptive to advice about children’s sun protection. It also found that parents were far less vigilant about their own sun protection than their children’s. A similar phenomenon was reported in an Australian thesis study [20].

Hamilton et al. conducted focus groups with parents in Australia [21], finding that they were well-equipped with sun protective knowledge, applying this in practice to varying degrees throughout the year. Parents associated numerous advantages with children’s protection, including health benefits, the instilment of positive sun safe attitudes, and parental peace of mind. Disadvantages included the loss of sun exposure benefits such as vitamin D absorption. Although parents described general societal approval regarding sun protection in children, often other adults or family members would make this difficult to achieve by setting a poor example through their own protection.

Although qualitative research with parents is limited, research has been carried out amongst other demographic groups, including university students [22, 23], female adults [24], and female adolescents [25]. A systematic review of qualitative studies including both male and female participants from several countries including the UK has also been done [26]. A common finding is that risks of UV exposure are well known, but this knowledge alone is often insufficient to motivate safe practices. Other influential factors play a role, including a sense of ‘unrealistic optimism’, due to a low perceived susceptibility to skin cancer [22, 23, 26]. Several benefits associated with sun exposure, including the desire to be tanned, feeling healthier, and getting enough vitamin D, also act as barriers to sun protection. [22–26]. Sun protection tends to be adopted when abroad rather than in the UK, especially when on holiday or at the beach [22, 24, 26]. Relevant to this study, family members are often influential in sun protection attitudes and behaviours, with Mothers often taking the lead role [22, 24, 26]. This further highlights the importance of carrying out research within this group specifically.

The existing body of qualitative research with parents comes from Australia [20, 21]. Having the highest global incidence rate of melanoma [2], Australia has implemented widespread sun protection awareness campaigns such as the ‘Slip, Slap, Slop’ programme [27]. Given these facts, it is probable that certain differences will exist within a UK population, and conducting UK specific research with parents is therefore of value.

The risk of melanoma is more than twice as high in individuals with the fairest skin type (Fitzpatrick type 1, see Table 1) compared to those with light brown skin (type 4), and risk is again lower amongst individuals with darker skin types (types 5 and 6) [28, 29]. For this reason, existing qualitative research has used predominantly Caucasian samples and has thus been unable to consider the impact, if any, of ethnicity and skin type on children’s sun protection. The majority of public health messaging adopt a ‘one size fits all’ approach, despite the fact that 14% of the UK population are of non-white skin type [3, 30]. This leads to a degree of uncertainty, whereby individuals with darker skin types experience greater confusion about their risk of skin cancer [3]. Whilst it is reasonable to focus on those at greatest risk, melanoma can occur in any skin type and other groups should not be neglected. By including parents of non-white children in this study, it is hoped that public health campaigns might be better able to target this issue in the future.

**Table 1:** Fitzpatrick skin type classification [29]

Fitzpatrick skin type	Descriptor
1 – Pale white	Always burns, does not tan
2 – Fair	Burns easily, tans poorly
3 – Darker white	Tans after initial burn
4 – Light brown	Burns minimally, tans easily
5 – Brown	Rarely burns, tans darkly easily
6 – Dark brown or black	Never burns, always tans darkly

This study is, to the best of the authors’ knowledge, the first UK based qualitative interview study investigating parents’ sun protection of their children. It aims to explore, amongst UK parents: (1) the degree of knowledge and understanding of sun protection in children, (2) the specific factors which motivate and challenge parents to adequately protect children, and (3) the impact of ethnicity and skin type on parents’ knowledge, attitudes and beliefs. A more thorough understanding of the factors that guide and influence parents’ sun protection for children can then be used to underpin future health campaigns by highlighting key messages which should be targeted.

## Methods

### Participants and recruitment

**Fig. 1:**
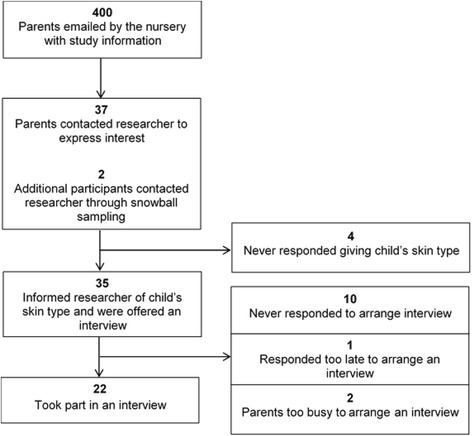
Flow of participants through the study

This qualitative study took part in Birmingham using face-to-face individual interviews with parents of children aged five or younger. This age was specified because younger children are most dependent upon parents’ protection. Figure 1 illustrates the flow of participants through the study. Parents (mothers or fathers) were recruited from a convenience sample at the Oaks and the Elms day nurseries at the University of Birmingham, each of which has 200 children enrolled. These nurseries were chosen due to proximity to the university campus and the high number of staff members who use them, so that inconvenience would not be a significant barrier to participation.

Nursery administrators distributed participant advertisements and information sheets to all parents via email. Those who expressed interest in participation were asked to classify their children’s skin type, using the Fitzpatrick skin type classification as shown in Table 1.

Of those who responded to this question, interviews were initially arranged on a first come first served basis. In an attempt to increase the number of participants with children of skin types 4–6, a snowball sampling method was used, inviting existing participants to pass on study information to parents who fulfilled these criteria [31]. The aim of this system was to enable purposive recruitment of equal numbers of parents of children with white (classified as type 1–3) and non-white (classified as type 4–6) skin types. This aim was not achieved, due to reasons which are discussed later in this paper.

### Data collection

A total of 22 individual interviews were conducted, taking place in private rooms at the university (*n* = 17) or in participants’ homes (*n* = 5). Written informed consent was obtained from all participants. In order to ensure participant anonymity, all were assigned numbers and gender specific pseudonyms which are presented alongside quotes. Interviews ranged in length from 9 to 32 min, and were conducted by ZL, a medical student at the University of Birmingham undertaking an intercalated BMedSc in Public Health and Population Sciences. All participants were aware that the interviewer was a medical student conducting research, and one participant knew the researcher through teaching at the medical school. Participants were accompanied by children for four interviews. Following completion, participants were given a Cancer Research UK ‘SunSmart’ information leaflet [32], and a £10 voucher to thank them for their time. Theoretical data saturation was reached by the end of the interview period, meaning that additional transcripts did not generate new themes, but essentially added to the depth of data obtained in previous interviews [18].

Individual interviews were chosen over focus groups as they enable participants to provide more detailed insights into less explored topics such as this one [33]. A semi-structured format allowed the interviewer to ensure that key topics were covered whilst also having flexibility to discuss issues that the participant introduced independently [34].

Interviews followed a topic guide, which is available in an Additional file 1. The topic guide was underpinned by a literature review and the components of the Health Belief Model [35]; a framework used to systematically consider concepts which influence why individuals may or may not engage in certain health behaviours [36]. In this case the Health Belief Model is applied to an adult adopting behaviours on behalf of their children.

A pilot interview was carried out to establish the approximate interview length, ensure the clarity of questions and ascertain the need for further probes. The topic guide was modified as interviews were conducted on the basis of reflexive practice and interview duration. All interviews were audio recorded and transcribed verbatim by the interviewer, ZL.

The nature of qualitative methods means the researcher has the potential to influence data collection and analysis [37]. A reflexive approach was maintained throughout the research process to enable recognition and management of this [37]. Field notes in the form of a research journal supported this process [38].

### Data analysis

Data was analysed following collection using Braun and Clarke’s six-phase guide to thematic analysis [39]. A grounded, data led approach was taken, allowing the themes to arise from the data itself rather than constraining the data into pre-specified categories [39]. ZL conducted and transcribed all interviews, and manually coded all transcripts. SG (medical sociologist), independently coded and analysed three of the transcripts.

Coded data extracts from all interviews were collated into a single document and organised into overarching categories, thus enabling better visualisation of potential themes and sub-themes. At this point the researchers met to compare analyses, and, following a discussion, agreed upon key themes. Thematic maps were produced to further refine these themes and visualise the connections between them. Finally, each theme and its accompanying data extracts were analysed independently, defining more precisely what each theme and sub-theme encompassed and revealed about the data [39].

## Results

Twenty-two mothers (*n* = 17) and fathers (*n* = 5) were interviewed, five of whom had at least one child with type 4–6 skin. The age of participants ranged from 29 to 42 years, with an average age of 36.5. Participants were asked about their occupation to provide context only and this was not used in analysis. Participants occupations are not listed due to confidentiality. They worked in a range of managerial and professional occupations reflected by categories 1 and 2 of the National Statistics Socio-economic Classification [40], and 13 worked in a university setting.

**Fig. 2:**
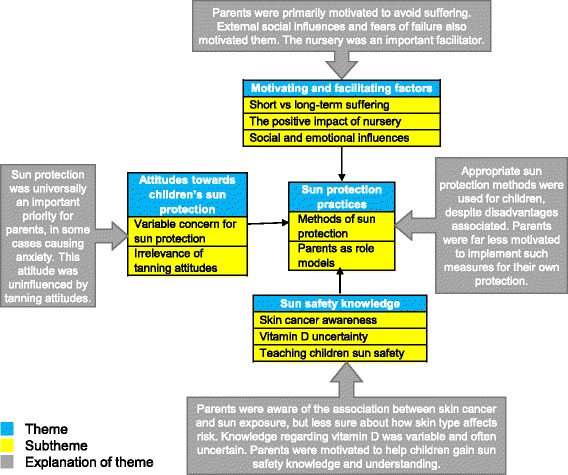
Diagram of themes and subthemes

Four main themes emerged: attitudes towards sun protection in children, sun protection behaviours, sun safety knowledge, and motivating and facilitating factors. Themes are broken down into sub-themes, which are shown alongside brief explanations in Fig. 2.

### Attitudes towards children’s sun protection

#### Variable concern for sun protection

All parents, regardless of skin type, felt that protecting their children from the sun was important and took appropriate precautions. The degree to which parents prioritised sun protection varied, with some being very concerned about sun protection, and others moderately so. For some, sun protection was a significant cause of anxiety:

“No, we haven’t [taken him on holiday abroad] yet and I’m petrified because of how fair his skin is. I would like to erm we’ve got two holidays in the UK booked for May and I’m really worried about them, erm but yeah no I’m petrified of taking him out of the UK” (Caroline).

Parents who were more concerned about sun protection perceived themselves to be extremely vigilant as a result. Some parents felt they might actually be overprotective:

“So I take it really seriously. A bit too seriously perhaps sometimes. But yes it’s really important to me” (Joanne).

#### Irrelevance of tanning attitudes

Parents had variable attitudes towards tanning. Some suggested that a positive attitude towards tanning was ingrained within them:

“He probably does, I suppose look healthier [with a tan] but I think that’s based on what we’re all conditioned to think is the case as opposed to he actually is healthier, so...” (Isobel).

Despite variable tanning attitudes, parents would not actively seek for their children to become tanned as they might themselves. In some cases, tanning was considered a natural consequence of time spent outdoors in summer, even when children were protected. Where positive tanning attitudes did exist, these did not inform how children were protected, as they were outweighed by knowledge of the risks of sun exposure, making tanning an unimportant factor:

“I’d rather him be healthy and safe than have a brown glow, he’s a small child” (Lisa).

### Sun protection practices

#### Methods of sun protection

Almost all parents referred to use of a combination of sun protection methods, primarily factor 50 children’s sunscreen, sun hats, covering the skin with clothing and limiting time in the sun. Some also used specialised UV protective equipment including full-body swimsuits, tents and pushchair shades.

Parents were more vigilant about sun protection when abroad compared to in the UK. This was because hot days in the UK are less frequent, and cloud cover was thought to offer some protection. Consequently, use of sun protection is less inclined to become part of the daily routine:

“I think when we’re on holiday I’m more conscious of it and I will top it up more regularly erm, but when you’re at home unless it’s really really hot and they’re outside all day I tend, I just tend to forget to be honest because you’re at home” (Louise).

Parents discussed disadvantages associated with certain types of sun protection. Despite being the most commonly referred to method, some parents felt that sunscreen was problematic in that it was physically unpleasant to use:

“Sun cream is thick and sticky so if you’re reapplying out and about it’s a nightmare because it gets everywhere and stains everything” (Caroline).

Three parents of children with darker skin types described a key issue associated with sunscreen being its discoloration of the skin:

“My daughter’s skin tone’s slightly darker than his, and then it’s like white on top of it so it gives like a bluey appearance. So she sort of looks a bit poorly when she’s wearing it” (Sarah).

Many parents discussed what they would look for when buying sunscreen. Some mentioned the need to ensure UVA and UVB protection and a five-star rating. For others, the meaning of these things was unclear:

“I mean I don’t understand when you buy the bottles of sun cream, all the different things that it says, UVA UVB all of these different things you’ve got to choose from” (Liz).

Some parents expressed a lack of trust in sunscreen and a sense of uncertainty regarding its standards, for example how the sun protection factor is proven. Furthermore, parents lacked confidence in properties such as all-day protection or water resistance:

“The newer creams say it will last for 8 hours. I don’t know how true that is, I suppose they have to put them through tests and things but that to me, that sounds too good to be true” (Catherine).

#### Parents as role models

Some parents recognised the fact that their children would often replicate their own behaviours and were conscious of the need to act as a positive role model for sun protection:

“Well I think as she, as she gets older she sees me putting it on, she doesn’t mind quite so much putting it on herself” (Liz).

Despite this, many parents reported far less vigilant behaviour for themselves:

“Erm but I could definitely improve how I look after myself. I would never let my kids treat their skin the way I treat may own” (Louise).

Different parents offered various explanations for this discrepancy in standards. Reasons included a lack of time, the desire to be tanned, and believing that their skin was less vulnerable than their children’s:

“I make the assumption that her skin’s more vulnerable and less well formed than it would be, than an adult’s skin. So we make more of an effort to protect her” (Matthew).

### Sun safety knowledge

#### Skin cancer awareness

Parents were well informed about the link between sun exposure and skin cancer, most citing it as a major motivation for their children’s protection. The media seemed to be partly responsible for this awareness:

“But there does seem to be quite a preoccupation with [skin cancer] in the media and sort of health, and saying it’s important. So I guess yes that must have an impact” (Mary).

Parents were asked whether they felt that their children’s skin type affected their risk of skin cancer. Responses to this question varied considerably. Some correctly understood that darker skin conferred lower risk for skin cancer [28]. Many were uncertain but speculated that because their child was fair and burnt easily, this meant that they might be at increased risk. Others felt that skin type did not influence their risk of skin cancer:

“I mean I don’t really know but I think skin’s just skin isn’t it at the end of the day” (Sarah).

Of parents whose children had skin types 4–6, two felt that this did not affect their risk, whilst three believed that their risk was reduced.

#### Vitamin D uncertainty

Parents described various advantages associated with sun exposure, including playing outdoors, building up tolerance to the sun, and mental well-being. The most commonly mentioned was the need for vitamin D. This made it challenging in some cases to achieve a balance and know when protection was warranted:

“Hearing the fact that children do need more vitamin D as well as you know having sun tan cream it’s quite a difficult balance to get, so I don’t really know the answer” (Isobel).

#### Teaching children sun safety

For many parents, educating children about sun safety was an important aspect of their sun protection. It was hoped that this would enable them to establish positive habits which children would continue as they grew up:

“I would hope that by being taught when they’re younger about the risks of the sun that they will remember it and be more likely to be vigilant when they’re older” (Gary).

Education was therefore a key motivational factor, however there were barriers to this. Most notable was the difficulty of understanding the concept of sunburn when most children had never experienced it:

“I think it’s difficult without having burnt to know quite what the, for them to sort of understand really what...” (Rose).

Generally, children who understood the basis of sun protection were felt to be more cooperative with sun protection, making it easier for parents:

“Yes my older child does understand it [...] He certainly would ask for sun cream to be put on, he probably wouldn’t ask to wear a hat or understand that covering his arms with clothes was helpful but he would come and ask for sun cream if he wanted to go outside” (Gemma).

### Motivating and facilitating factors

#### Short vs long-term suffering

Parents were motivated by both the long-term risks of sun exposure and the short-term risk of sun burn leading to suffering:

“…you know damn well that if you don’t do it they are going to experience pain. So, you know, there’s a very good immediate reason to do it, with or without a longer-term reason” (Daniel).

Despite the fact that darker skin burns less quickly than paler skin, short-term motivation still existed for parents whose children had darker skin types:

“Despite the fact that he is of colour erm he you know still needs that protection. I protect my skin so you know, I’ve burnt in the sun before so I know that erm if that can happen to me it can obviously happen to him” (Megan).

#### The positive impact of nursery

Children’s sun protection was facilitated by the nursery, who fulfilled three important roles. Firstly, by asking parents to provide sunscreen and hats, the nursery served as a reminder to parents and helped them to develop a sun protection routine, which was often continued at home. Katie explained this when asked where she learnt about children’s sun protection:

“I guess partly just kind of general knowledge but also practices that they use at nursery as well when they’re young so I guess mimicking some of the stuff they do there, so they’re always very diligent about sun hats and sun cream and things so...” (Katie).

Secondly, nursery also helped to normalise the use of sun protection amongst children, since it was something which they all did together as a peer group:

“It’s teaching the children that you have to protect yourself from the sun and because the other children they will do it as well, they don’t find it as a hassle that you then do it and they ask for you to do it” (Anna).

Thirdly, the nursery also played an educational role, with many parents believing that their child’s understanding of sun protection had largely arisen from their experiences at nursery:

“So they always, they tell why, so I think that she got it from nursery, to the fact that when it’s sunny and it’s warm and you go outside, you put sun cream on, because it protects you from the sun.” (John).

#### Social and emotional influences

Sun protection was viewed as a parental duty. Many participants described a sense of guilt or failure that they would feel if their child got sunburnt. Avoiding this guilt was clearly an important motivation:

“I’d be devastated, yeah I’d be really upset because I’d think it was a failing on my part. But obviously with the age that he is erm he’s reliant on myself on my husband to protect him, so erm yeah I’d feel, I’d feel pretty awful about it yeah.” (Megan).

Peer groups such as friends and family were also identified as influential factors. Some parents were concerned about what others might think if their child was sun burnt or tanned. Some commented that within their social groups, tanned children would not be looked upon favourably:

“People that I spend time with and the parents that I socialise with, their kids are never ever brown […] so I guess there’s a bit of a sense of what might people think if you weren’t taking care of this, you know” (Joanne).

## Discussion

These findings provide insights into the practices adopted by parents in protecting children from the sun, their sun safety knowledge and attitudes that inform and influence these behaviours, and factors which motivate and facilitate them in doing this. Sun protection in children was universally an important matter for the parents in this study, who had good knowledge of appropriate precautions and adopted these in practice for their children. This is especially the case when parents’ are abroad, echoing findings from previous studies in other groups [22, 24, 26].

Although findings from this sample cannot be extrapolated to all parents, the diligence of these parents’ sun protection is generally dissonant with the fact that the incidence of skin cancer in the UK is rising [2]. It is possible that other social and cultural factors such as sun protection practices later in life, the ageing population and increasing foreign travel are more important causative factors in this trend. Nevertheless, certain areas were identified in this study which might have the potential to further improve sun protection early in life.

It can be useful to consider findings from qualitative research in the context of pre-existing theory [41]; in this case using the Health Belief Model (HBM) [35]. The HBM states that an individual is inclined to take health protective behaviour (in this context sun protection), if certain criteria are fulfilled. Firstly, parents must perceive that children are susceptible to the condition (skin cancer), and that the condition would have serious consequences [36]. Although the severity of skin cancer was not discussed specifically in this research, perceived susceptibility was implied as skin cancer prevention was a major motivation for implementing sun protective behaviours.

Perceived benefits of a health behaviour must outweigh the barriers [36]. In this case, a number of benefits to sun protection were discussed. Some related to health outcomes; namely reducing the risk of skin cancer and sunburn in children. Other reasons included teaching children good habits, reducing guilt in parents, and ensuring social approval. These findings are consistent with sun protection benefits identified by parents in Australian qualitative research [21].

Several barriers to sun protection related specifically to sunscreen. These included a perceived unpleasantness associated with it amongst some parents, its tendency to discolour darker skin, difficulty trusting the properties that it claims to have, and uncertainty about sunscreen labelling.

Vitamin D was commonly cited as a benefit of sun exposure, echoing findings from previous qualitative research [23, 24, 26]. As a result, participants experienced difficulties in making decisions regarding vitamin D requirements and the need for sun protection, especially when in the UK. These findings are consistent with previous research, in which participants were confused by conflicting messages regarding vitamin D and sun protection, and thus achieving this balance was challenging [42, 43].

Previous research has highlighted how a desire to be tanned can influence personal choices about sun protection [22, 25, 44]. Conversely, in this study it was found that parents’ tanning attitudes did not appear to influence or act as a barrier to their children’s sun protection. Protecting children from the harms of UV exposure was the main priority amongst parents, and none in this study were motivated to get their children tanned. Despite this, some parents considered tanning to be an inevitability in summer, even with appropriate protection. This was especially the case amongst children with darker skin types which tan more easily [29], in which cases tanning was not necessarily viewed as a harmful or negative outcome.

Parents often adopted lower standards for their own sun protection, making them poor role models for their children. This finding echoes results from existing qualitative research [19, 20]. Although this was not identified as a barrier by parents, research has demonstrated that this has a negative effect on children’s sun protection. Use of sun protection in parents is a predictor of sun protection in children [45, 46], and sunburnt parents are more likely to have sunburnt children [10]. This discrepancy in standards may be due to the principle of ‘unrealistic optimism’ as identified amongst adolescents and adults in previous research [22, 23, 26], whereby parents don’t perceive themselves to be at serious risk of skin cancer, whilst children are viewed as being more vulnerable to the harmful effects of the sun.

Modifying variables are individual factors, such as ethnicity, which indirectly influence perceptions of health behaviours and outcomes [36]. Although skin type was considered throughout the analysis, too few participants with type 4–6 skin were recruited to gain sufficient data to answer this research question and no difference was observed between these parents and the rest of the participants in terms of sun protection concern, motivation and methods. Skin type was seen to influence perceived susceptibility to cancer in some cases, although there was some uncertainty regarding this issue.

Self-efficacy refers to an individual’s confidence to take action [36]. In this research, parents were mostly confident and well informed about sun protection. For some parents, sun protection was a cause of fear and anxiety, suggesting that their sense of self-efficacy was lower.

A cue to action is something which can help to trigger a health behaviour [36]. In this study, the nursery could be thought of as such, as their sun protection policy helped to remind parents and aided them in developing sun protective habits. The nursery also provided wider benefits, helping to educate children and normalise use of sun protection. The benefit which childcare facilities can have in sun protection has previously been recognised, and as such current UK guidelines recommend that all educational environments implement a sun protection policy [8].

### Recommendations

The findings from this study have brought to light a number of key areas where uncertainty or challenges exist for parents in children’s sun protection. More widespread and comprehensive guidance for parents may not only benefit those who do not provide adequate protection, but also provide reassurance to those for whom their children’s sun safety is a considerable source of concern and anxiety.

Specific areas which should be highlighted in future campaigns include increasing awareness of the need for sun protection in the UK as well as when abroad. Further sunscreen guidance may also be helpful, by increasing awareness of important features of labels including the need for adequate sun protection factors and high star ratings in order to ensure complete protection [47].

This study, along with previous research, has highlighted a problem with parents’ own sun protection [19, 20]. Future campaigns might be more effective if targeted towards the family as a whole, in order to encourage not only the importance of protecting children from the sun, but also the benefit that adults as positive sun safety role models can have.

Given the increasing prevalence of the issue of vitamin D in the media throughout the past decade [48], and the resultant uncertainty which many parents express about this, it may be beneficial for future public health campaigns to target this in greater detail. For example, increasing awareness of the use of UV ratings may prove to be a useful indicator to parents of when sun protection is warranted [32].

The influence of the nursery in this study serves as a good example for the benefit that an effective sun protection policy can have in educational establishments. Existing research suggests that sun protective behaviours typically decline with progression through primary school [49]. A qualitative study focussing on parents of primary school age children would be an interesting and valuable subject of future research to investigate this phenomenon and its possible causes. Ensuring that all nurseries and schools implement effective and comprehensive sun protection policies may help to establish positive behavioural habits in childhood and enable their continuation through adolescence into adulthood.

### Strengths and limitations

This is, to the best of the authors’ knowledge, the first UK based qualitative interview study to investigate parents’ knowledge, attitudes and beliefs regarding sun protection in children; an important topic considering the increasing prevalence of melanoma skin cancer in the UK, and the vulnerability of the skin during childhood and adolescence [5–7]. Sufficient interviews were carried out to enable data saturation to be reached [18].

Steps were taken to ensure methodological rigour. The researcher followed a pre-defined structure of data collection and analysis (Braun and Clarke’s six phase guide to thematic analysis [39]) which was not deviated from. All steps of this process were demonstrable in the form of an audit trail. Credibility of results was ensured through triangulation of analysis, whereby a second researcher independently coded and analysed several transcripts, and both researchers reached agreement about the conclusions which could be drawn from these [37]. Furthermore, a reflexive approach was adopted and supported by the use of a research journal throughout the process. This facilitated recognition of ways in which the researcher may have influenced data collection or analysis [37].

The sampling and recruitment strategies used in this study are likely to have had an impact on the data collected. The majority of participants were employed at the university and worked in managerial or professional occupations, and thus not representative of all parents. Given those of higher socio-economic status generally adopt healthier behaviours [50], it is probable that participants’ backgrounds impacted upon children’s sun protection. There was also a gender imbalance as most participants were Mothers, whose beliefs and attitudes may differ from those of Fathers. It is possible that the findings of the study would have been different had there been more variation with respect to these characteristics.

The majority of participants in this study were from the same nurseries, which were seen to positively influence children’s sun protection. It is possible that a study conducted with parents from another nursery could therefore differ. Whilst it is important to acknowledge these limitations, the goal of qualitative research is not to produce generalisations but to provide a rich understanding of individual cases in their own unique context through in-depth discussion and analysis [51].

The study aimed to recruit equal numbers of white and non-white participants. This was not achieved due to a number of factors including: a lack of initial interest from non-white participants, limited response to snowball sampling attempts [31], and no responses from other nurseries contacted for recruitment purposes. There was therefore limited opportunity to explore the impact of skin type and ethnicity on parents’ knowledge, attitudes and beliefs. More extensive research with greater capacity to recruit these participants is needed.

Participants were aware that the researcher conducting the interviews was a medical student. This may have created a social desirability bias, whereby responses were adapted to suit what a participant felt the interviewer, as a future health professional, might want to hear, or what the participant felt was socially desirable [52].

## Conclusion

Parent’s in this qualitative research, regardless of children’s ethnicity, were found to be well equipped with knowledge of sun protection methods, and motivated to apply this knowledge in protecting their children. It identifies key areas of uncertainty such as vitamin D needs, sunscreen properties and the need for protection in the UK, which might be targets of future campaigns. Potential areas for future research have also been identified and highlighted.

Whilst it is appropriate that sun protection is a priority for parents, it should not be a significant source of anxiety. Clearer and more widespread sun protection guidance focussing on specific areas of uncertainty might reduce this anxiety, as well as increasing sun protection knowledge and confidence amongst parents generally.

## Additional file

Additional file 1: Topic guide. (DOCX 14 kb)

## Abbreviations

HBM: Health Belief Model
UV: Ultraviolet
