# Supplementary material for: Parents’ knowledge, attitudes and beliefs regarding sun protection in children: a qualitative study
Source: BMC Public Health. 2018 Feb 1;18:207. doi: 10.1186/s12889-018-5091-8 (PMC5796497; doi:10.1186/s12889-018-5091-8)
Supplement: Additional file 1: — Topic guide. (DOCX 14 kb) [file 12889_2018_5091_MOESM1_ESM.docx]

**Topic Guide**

Introduction

- Clarify number, ages and gender of children
- Confirm children’s skin type using images

Exploring knowledge and behaviour

- How important is it to you to ensure that your son/daughter is protected from the sun?
- What sun safety practices do you tend to use?
  - *Probe:* Why are [these methods] preferred over others?
- Do you consider sun exposure to be a risk throughout the year?
  - *Probe:* How do your sun protection practices change accordingly?
- Do you travel to warmer countries for holiday?
  - *Probe:* How do your sun protection practices change if so?
- What do you consider to be the most effective way to protect children from the sun?
- How do you as a parent learn how to best protect your child from the sun?
- Does your child understand sun protection?
  - *Probe:* How do they learn this?
  - *Probe:* If not, at what age?

Beliefs about sun protection

- What motivates you the most from day to day in protecting your son/daughter from the sun?
  - *Probe:* What risks of exposure are considered most significant? 🡪 Sunburn/ageing/skin cancer?
  - *Probe:* Do you consider skin cancer as a serious risk for your child?
- In what way do you think your child’s skin type affects their risk of skin cancer?
- Do you think the effort/burden of sun protection in young children always outweighs the risks of sun exposure?
- Are there any disadvantages which come with different ways of protecting children from the sun?
- Has your child ever been sunburnt?
  - How did it/would it make you feel as a parent?
- Do you think your child would look healthier if they came back from a holiday with a tan?

Parent as a role model

- Do you take the same amount of care in protecting yourself from the sun as you do your son/daughter?
  - *Probe:* If not, why not?
- Does your attitude towards getting a tan on holiday differ for yourself and your son/daughter?

Barriers to good sun protection

- Do you generally feel confident in your ability to effectively protect your son/daughter from the sun?
- What are the biggest challenges that you face in ensuring your son/daughter is protected from the sun?
- Is there anything that you think could be done which might help to improve sun protection in children or make it easier for parents?

Thank the participant
